# Novel Bioderived Composites from Wastes

**DOI:** 10.3390/ma13112571

**Published:** 2020-06-05

**Authors:** Andrea Petrella, Marco Race, Danilo Spasiano

**Affiliations:** 1 Department of Civil, Environmental, Land, Building Engineering and Chemistry, Polytechnic University of Bari, via E. Orabona, 4, 70125 Bari, Italy; danilo.spasiano@poliba.it; 2 Department of Civil and Mechanical Engineering, University of Cassino and Southern Lazio, Via di Biasio 43, 03043 Cassino, Italy; marco.race@unicas.it

## 1. Introduction

The recycling and reuse of solid wastes can be considered important challenges for civil and environmental applications in the frame of a more sustainable model of development and the consumption of new resources and energy [1,2,3,4,5]. The recovery of raw materials from nonconventional sources and their transformation into usable resources not only represents an economic advantage, but also offers an ecological opportunity for the utilization of by-products which would otherwise be landfilled [6,7,8,9,10,11]. In this respect, these secondary raw materials, generally derived from industrial, agricultural and food manufacturing activities, become an abundant resource that can be easily reused for different applications, as reported in the recent studies collected in this Special Issue. 

For that purpose, six papers were related to the preparation of innovative composite materials. Specifically, five papers reported the reuse of end-of-life tire rubber, porous glass, expanded polystyrene, slags, fly ashes and sheep’s wool fibers for the preparation of cement conglomerates [12,13,14,15,16], while the last one reported the reuse of amorphous silica nanoparticles for the preparation of composites with natural rubber [17]. Moreover, five papers were related to the treatment of wastes for environmental applications. Specifically, two papers reported the reuse of egg by-products [18] and crab shell [19] for the removal of biopersistent micropollutants, and two papers reported the reuse of white bamboo fibrils as oil absorbent [20] and a rapid method for the disposal of radioactive contaminated soil waste [21], respectively. The last one reported the use of wheat straw biochar for cobalt sorption from contaminated soil [22].

## 2. Waste Products for Construction Materials

In the papers by Petrella and coworkers [12,13], recycled materials, such as end-of-life tire rubber (TR), porous glass (PG) and expanded polystyrene (EPS) were used as aggregates for the production of unconventional cement mortars. A cheap and environmentally safe process was employed, since no pre-treatment of the renewable aggregates was carried out. The thermal conductivity of these lightweight composites was 80–90% lower than the conventional sand mortars. Moreover, the presence of the recycled glass (PG) influenced the mechanical strengths and the thermal insulation of the specimens, thanks to the high stiffness and closed porosity of the aggregate [23], while the conglomerates with end-of-life tire rubber and expanded polystyrene showed thermal insulation and hydrophobic behavior due to the low water absorption. These results revealed that these composites may be suitable for nonstructural thermo-insulating products, specifically for the production of inside and outside elements.

Perez-Garcia and coworkers studied the properties of green cementitious grout mixtures characterized by cement substitution with slag (25% and 50%) derived from steel manufacturing [14]. The addition was carried out without additives and the slag was introduced as a cement replacement. Specifically, different slags (ladle furnace slag (LFS) and blast furnace slag (GGBS)) were used for the preparation of cheap conglomerates, which were tested for exudation, compressive and flexural strength, in order to analyze the feasibility of the mixtures for industrial applications. In general, these conglomerates showed a lower density and an improvement in fluency and viscosity with respect to the conventional references, while the mechanical response was dependent on the origin of the slag. The fluidity of the mixtures allows for their use in applications such as jet grouting or ground improvements. GGBS slags improved the mechanical strengths and workability of the mixtures, while the LFS slags can be employed in other types of works where a high strength is not required. 

The paper authored by Wei-Ting Lin and coworkers [15] showed the feasibility of using ground-granulated blast-furnace slag (S) and circulating fluidized bed co-fired fly ash (FA) as non-cement binding materials. In fact, they determined the optimal mix proportions (100% cement replacement, S:FA ratios of 4:6, 5:5, 6:4, water/binder ratio of 0.55) in order to achieve high dimensional stability and good mechanical properties with inclusion in the resulting composite of polypropylene fibers. Composites with an S:FA ratio of 6:4 showed a compressive strength approximately equal to 30 MPa, which is 80% the strength of conventional cement-based materials at 28 days of curing. The strong influence of the polypropylene fibers in reinforcing the non-cement blended materials was demonstrated, indeed the inclusion of 0.2% fibers in the mixture further increased the compressive strength to 35 MPa and also enhanced the compactness of the micropore structures, increased the tensile strength and decreased absorption and the likelihood of shrinkage. 

In the paper by Maia Pederneiras and coworkers [16] sheep’s wool fibers were incorporated into mortars to ensure the durability of the render and improve the flexural strength, fracture toughness and impact resistance. The novel composites were prepared with cement and cement–lime ligands. The addition of 10% and 20% (in volume) of 1.5 cm and 3.0 cm wool fibers led to the increase in the ductility of the mortars and an improvement in the mechanical properties. In fact, these specimens showed high ductility because they presented a higher flexural and compressive strengths ratio (σf /σc) with respect to the reference mortars. The conglomerates also showed an improvement in the fracture toughness, with specific reference when longer fibers were incorporated. Moreover, the presence of longer fibers affected the increase in the flexural and compressive strengths. The wool fiber composites also presented a lower tendency to crack when compared with the conventional artifacts.

Nguyen and coworkers reported a method of recovering amorphous silica nanoparticles (40–60 nm) from hexafluorosilicic acid waste (Vietnamese fertilizer industry) through a precipitation process [17]. These particles were adopted as a reinforcing filler of natural rubber (NR) materials, which were characterized by morphological, mechanical, rheological and thermal measurements. Specifically, the mechanical properties of nanosilica-filled NR composites reached the optimum with 3 phr of nanosilica, and accordingly the tensile strength, hardness and decomposition temperature of these novel materials showed an improvement of 20.6%, 7.1%, and 2.5%, respectively, with respect to the pristine NR. The hardness of the filled samples increased with increasing nanosilica content, as opposed to the elongation at break. The improved mechanical properties can be explained by the tensile fractured surface morphology, which shows that the silica-filled NR is rougher than the pristine natural rubber sample. 

## 3. Waste Materials for Environmental Science

A reusable adsorbent constituted by eggshell was proposed by Murcia-Salvador and coauthors for the removal of Direct Blue 78 (DB78) dye from wastewater [18]. Notably, the maximum adsorption of DB78 onto eggshell was obtained at pH 5 and 12.5 g/L of adsorbent dosage and the adsorption capacity of DB78 was 13 mg/g. The study of the thermodynamic parameters highlighted that the adsorption process was endothermic and spontaneous in the 29–75 °C range. In addition, the combination of the adsorption process on eggshell and the H_2_O_2_/pulsed light advanced oxidation process led to a further decrease in the pollutant concentration, thus demonstrating that the adoption of both processes can be used successfully in the removal of dyes at higher concentrations from wastewater.

The paper contributed by Rizzi and coworkers introduced the use of chitosan from biowaste (crab shell) to induce the formation of solid films useful for the decontamination of water from emerging pollutants [19]. In particular, ketoprofen was used as a contaminant, and a high percentage of removal, at least 90%, was shown in a short time under the proposed experimental conditions. Moreover, the authors detailed the nature of the adsorption by changing the chemical and physical parameters, such as the pH, temperature changes and electrolyte presence in the solutions containing the pollutant. The interaction between the ketoprofen carboxylic moiety and the chitosan amino groups were proposed by showing that the presence of salts inhibited the adsorption process, giving the opportunity to desorb the pollutant and recycle both the adsorbent material and ketoprofen. 

Nguyen and coauthors reported the synthesis of highly porous cellulose aerogels, produced from white bamboo fibrils, which could be adopted to clean up oil spills and toxic chemicals in aquatic environments [20]. Specifically, white bamboo was cut and placed into an autoclave for 60 min. Afterwards, samples were immersed in a 2% NaOH solution at 70 °C to remove the cell walls and the obtained fibers were ground until they reached a micron-sized diameter. These cellulose fibers were dispersed in a NaOH/urea/H_2_O mixture, leading to a cellulose hydrogel, which was washed with water and then freeze-dried. Finally, MEMO silane was deposited on the cellulose-based aerogel. This silane-treated cellulose aerogel exhibited high absorption capacities of 1091 ± 19.6%, 1237 ± 17.6% and 1247 ± 21.1% by weight gain for waste motor oil, diesel and gasoline, respectively.

Xue and coworkers described a rapid and effective method for the disposal of radioactive contaminated soil waste [21]. For this purpose, simulated Ce-bearing radioactive soil waste was immobilized by the self-propagating high-temperature synthesis (SHS) of forms containing 5–25% of contaminated material and which were characterized by the analysis of phase composition, microstructure and chemical durability. The simulated nuclide Ce was immobilized into a pyrochlore-rich waste matrix characterized by multiphase composite materials (SiO_2_, Gd_2_Ti_2_O_7_ and Cu). Moreover, it was observed that the simulated nuclide Ce was simultaneously present in the pyrochlore and soil phases, thus indicating a partial migration of Ce during the SHS reaction. The solidified body of a Cu-20 sample (with 20% of soil waste) exhibited high stability. 

Finally, Medyńska-Juraszek and coworkers used wheat straw biochar for cobalt sorption from contaminated soil [22]. It was demonstrated that this material was an efficient sorbent, decreasing the mobility and availability of Co^2+^ in soil and reducing health risks related to human exposure. The dominant mechanisms of sorption were mainly associated with interactions with carboxylic and hydroxyl groups present on the biochar surface. Cobalt immobilization was more complex because the efficiency of the process can be modified by biochar oxidation and interaction with soil constituents. 

## 4. Outlooks

The above-mentioned papers have demonstrated that the recovery of solid wastes from industrial, agricultural and food manufacturing activities can be considered an important challenge for the design of new materials and for the evolution of new techniques in the frame of a more sustainable model of development and the consumption of new resources and energy.
